# Effects of Elamipretide on Autophagy in Renal Cells of Pigs with Metabolic Syndrome

**DOI:** 10.3390/cells11182891

**Published:** 2022-09-16

**Authors:** Siting Hong, Ramyar Ghandriz, Sarosh Siddiqi, Xiang-Yang Zhu, Ishran M. Saadiq, Kyra L. Jordan, Hui Tang, Khaled A. Ali, Amir Lerman, Alfonso Eirin, Lilach O. Lerman

**Affiliations:** 1 Division of Nephrology and Hypertension, Mayo Clinic, Rochester, MN 55905, USA; 2 Department of Cardiology, First Affiliated Hospital of Harbin Medical University, Harbin 150001, China; 3 Department of Cardiovascular Diseases, Mayo Clinic, Rochester, MN 55905, USA

**Keywords:** kidney, metabolic syndrome, autophagy, mitochondria, inflammation

## Abstract

Autophagy eliminates excessive nutrients and maintains homeostasis. Obesity and metabolic syndrome (MetS) dysregulate autophagy, possibly partly due to mitochondria injury and inflammation. Elamipretide (ELAM) improves mitochondrial function. We hypothesized that MetS blunts kidney autophagy, which ELAM would restore. Domestic pigs were fed a control or MetS-inducing diet for 16 weeks. During the 4 last weeks, MetS pigs received subcutaneous injections of ELAM (0.1 mg/kg/day, MetS + ELAM) or vehicle (MetS), and kidneys were then harvested to measure protein expression of autophagy mediators and apoptosis. Systemic and renal venous levels of inflammatory cytokines were measured to calculate renal release. The function of isolated mitochondria was assessed by oxidative stress, energy production, and pro-apoptotic activity. MetS slightly downregulated renal expression of autophagy mediators including p62, ATG5-12, mTOR, and AMPK vs. control. Increased mitochondrial H_2_O_2_ production accompanied decreased ATP production, elevated apoptosis, and renal fibrosis. In MetS + ELAM, mito-protection restored autophagic protein expression, improved mitochondrial energetics, and blunted renal cytokine release and fibrosis. In vitro, mitoprotection restored mitochondrial membrane potential and reduced oxidative stress in injured proximal tubular epithelial cells. Our study suggests that swine MetS mildly affects renal autophagy, possibly secondary to mitochondrial damage, and may contribute to kidney structural damage in MetS.

## 1. Introduction

Obesity and its sequelae, such as metabolic syndrome (MetS), are urgent healthcare concerns. Their rise has been fueled by global increases in sugar and fat consumption, accompanied by reduced rates of physical activity [1,2]. Mets impairs various cellular mechanisms by fostering an inflammatory milieu and dysregulating adipokine and cytokine levels. Among vital processes affected by MetS [3], autophagy is an evolutionarily conserved mechanism through which cellular energy products are recycled and reutilized via lysosomal degradation of organelles [4]. This compensatory mechanism supports cellular function during aberrant nutrient availability, mostly via macro-autophagy and micro-autophagy, to reutilize cellular constituents and increase the efficacy of energy production and utilization [5]. Macro-autophagy is a response to environmental and physiological factors, through formation of a double-membrane autophagosome that then fuses to a lysosome to degrade cargo, such as targeted proteins and organelles [6,7]. Micro-autophagy is a non-selective degradative process that involves deformation of the lysosomal membrane to engulf cytoplasmic content such as useless proteins, especially during starvation [8,9]. Consequently, autophagy closely interacts with the cellular mitochondria, which are crucial elements in cell survival and metabolic regulation [10]. Autophagy has a central role in delaying formation of atherosclerotic plaques but might be impaired by MetS [11] or diabetes [12] and its restoration improves vascular function in aged mice [13].

Mitochondria play a pivotal role during times of increased environmental strain by regulating the endoplasmic reticulum (ER) stress response and modulating both autophagy and apoptotic signaling [14]. For example, in pro-oxidative conditions, mitochondria-associated ER membranes can transfer p66Shc and produce reactive oxygen species (ROS), which stimulates ER stress [15]. Oxidative stress can in turn stimulate mitochondrial-derived vesicles to transport selected cargo to the lysosomes, complementing mitophagy [16]. Autophagy is often activated in response to adverse environmental conditions, including nutrient deprivation or surplus, inflammation, or mechanical stress, when autophagy serves to restore cellular homeostasis. These stresses may increase ATP requirements, mitochondrial ROS production, and NLRP3 interactions with Beclin-1 [17,18]. Severe stress, however, can tip the cellular response from autophagy to apoptosis [19] and eventuate in cellular loss.

While the physiologic autophagic response is upregulated during cellular stress, it might be downregulated and rendered inefficient by several disease states, including those involving mitochondrial impairment [20,21]. Tissues and organs are heavily reliant on efficient mitochondrial and autophagic function is particularly susceptible to such forms of damage. For example, kidney tubular cells possess large numbers of mitochondria due to their role in active transport, an energetically demanding process [22]. They are also highly dependent on autophagy as a means of meeting their energy requirements efficiently [23]. Insufficient autophagy can cause the accumulation of dysfunctional and toxic organelles, making it fail in keeping cellular homeostasis of nutrients [24]. When mitochondria are unable to adequately regulate autophagy and apoptotic signaling, the kidney becomes increasingly prone to parenchymal injury and loss of function [25].

Obesity and MetS may also elicit mitochondrial dysfunction, structural damage, and increased rates of mitochondrial degradation [26]. This might be secondary to superfluous lipids, excessive renal tubular sodium reabsorption, and activation of the renin-angiotensin-aldosterone system (RAAS), leading to glomerular hyperfiltration and glomerulosclerosis [27]. Diabetic nephropathy involves hemodynamic changes, increased salt sensitivity, RAAS activation, and reabsorption of glucose, leading to increased ROS production and hyperglycemia, altering mitochondrial energetics [28,29]. This may result in renal tubular overload, increased proximal tubular sodium and water reabsorption, elevated ATP consumption and decreased production, and increased mitochondrial fission [29,30]. Previous studies have shown that angiotensin-II lowers mitochondrial membrane potential and depresses mitochondrial energy metabolism [31]. In addition, obesity inhibits mitochondrial fatty acid β-oxidation, damaging their cristae membrane and matrix density and inducing release of H_2_O_2_ that further damages the cell, while lipotoxicity dysregulates cell survival and recycling of damaged proteins, lipid droplets, and waste products [5]. We have previously shown that diet-induced MetS evoked in pig kidneys mitochondrial dysfunction, in association with decreased content of the mitochondrial inner membrane phospholipid cardiolipin [32,33,34], a key regulator of mitochondrial function and structure. However, its effect on renal autophagy is incompletely understood.

Elamipretide (ELAM, also known as SS-31, MTP-131, and Bendavia) is a specific mitochondrially targeted tetrapeptide (D-Arg-dimethylTyr-Lys-Phe-NH2) that binds selectively to and stabilizes cardiolipin by disrupting its interactions and oxidative inactivation by cytochrome-c [35]. ELAM thereby protects mitochondria from cellular stress [36], and we have demonstrated its ability to preserve mitochondrial structure and function and decrease oxidative stress [32]. Moreover, ELAM ameliorated renal hypoxia and injury in the context of MetS [25]. However, the effects of MetS on renal cellular autophagy, the involvement of inflammation and mitochondria in its regulation, or the ability of ELAM to restore it, remain unclear.

Therefore, this study was designed to test the hypothesis that autophagy would be blunted in a preclinical pig model of MetS and restored by mitochondrial protection with ELAM.

## 2. Materials and Methods

### 2.1. Animal Experiments

The Mayo Clinic Animal Care and Use Committee approved this study (A47613-13, 10/7/2013). Eighteen female domestic pigs (3 months old) were studied for 16 weeks. The animals were initially randomized into MetS (n = 12) and Lean (n = 6) groups. The MetS group had free access to a high-cholesterol/high-carbohydrate diet (17% protein, 20% complex carbohydrate, 20% fructose, and 43% fat) supplemented with 2% cholesterol and 0.7% sodium cholate (Purina Test-Diet, Richmond, IN, USA) [37]. The lean group was fed for the duration of the study standard pig chow containing 14.5% protein and 3% fat with 3.3 Kcal/g of feed (Purina Animal Nutrition, Shoreview, MN, USA).

After twelve weeks of diet, MetS pigs started a 4-week regimen of daily subcutaneous injections of ELAM (0.1 mg/kg; Stealth Biotherapeutics, Needham, MA, USA) in 1 mL phosphate-buffered saline (PBS) [38,39] or PBS vehicle only (n = 6 each). Fasting blood samples were ultimately collected to assess lipid levels, renal function, blood sugar, and insulin, and MetS assessed by calculating the homeostasis model-assessment of insulin resistance (HOMA-IR) score (fasting plasma glucose×fasting plasma insulin/22.5) [40]. The inferior vena cava (IVC) and right renal vein were then cannulated for blood collection and contrast media injections, and single-kidney renal blood flow (RBF) measured using computed tomography (CT) as described [25,32,41]. 

Upon the completion of the regimen, the pigs were euthanized with a lethal-dose injection of sodium phenobarbital. The kidneys were removed and immediately dissected, and sections were frozen in liquid nitrogen or preserved in a formalin fixative for ex vivo studies.

### 2.2. Autophagy Markers 

To evaluate autophagic activity in renal cells, we assessed by Western blotting protein expression of several central mediators: microtubule-associated protein 1A/1B-light chain (LC3) I/II, p62, 5′-adenosine phosphorylated-monophosphate-activated kinase (p-AMPK), mammalian target of rapamycin (mTOR), UNC-51-like kinase-1 (ULK-1), autophagy-related (ATG)-5-12 complex (all 1:1000, Cell-Signaling, Danvers, MA, USA), p-AKT, and ribosomal protein-S6. Glyceraldehyde 3-phosphate dehydrogenase (GADPH) (1:5000, Abcam, Boston, MA, USA) served for loading control [42], except for p-AMPK, p-AKT, and p-S6 proteins that were normalized to their total proteins. 

In addition, to evaluate for mitophagy, we stained for colocalization of PINK (1:100, sc-517353, Santa-Cruz, Santa Cruz, CA, USA) and Parkin (1:100, sc-32282, Santa-Cruz). The M1 factor of colocalization [43] was determined in 10–15 random fields in 5–6 samples per group.

### 2.3. Mitochondrial Energy Production and Cellular Apoptosis

To measure renal cell mitochondrial energy production and oxidative stress, renal mitochondria were isolated using a MITO-ISO kit (ScienCell, Carlsbad, CA, USA, #8268) [44]. Renal production of superoxide anion was evaluated by fluorescence microscopy using dihydroethidium (DHE) [45]. ATP, ADP, and mitochondrial hydrogen peroxide (H_2_O_2_) production were then calculated by colorimetric methods (Oxis, BIOXYTECH H_2_O_2_-560 Assay, #21024, and Abcam, #ab83355., respectively) [25] and ATP/ADP ratio calculated. Cytochrome-c oxidase (COX)-IV activity was assessed by fluorometric methods (Abcam; Cat#ab109909). The degree of renal cell apoptosis was assessed by staining kidney sections with terminal deoxynucleotide transferase-mediated dUTP nick-end labeling (TUNEL) (Promega, #G3250) and by calculating the ratio of renal mitochondrial to cytoplasmic cytochrome-c protein expression [41,46].

### 2.4. Fibrosis and Lipid Deposits

Kidney tissue embedded in paraffin was cut into 5µm sections for trichrome staining that was analyzed in 8-10 fields per slide (ZEN; Carl Zeiss, Oberkochen, Germany). The degree of interstitial fibrosis was quantified semi-automatically and expressed as the proportion of blue pixels of total area [41]. To assess renal lipid deposits fresh frozen renal slices were stained for Oil-red-O. The percentage of red to the total tissue area was measured by Image-J and averaged in 6 random fields/slide [33].

### 2.5. Inflammatory Biomarkers Levels

Renal vein and inferior vena cava (IVC) levels of soluble IL-1β, IL-6, IL-10, IL-18, IL-1α, and TNF-α were measured by Luminex (Millipore, Billerica, MA, USA). Then, renal net release was calculated by multiplying right-kidney renal blood flow (RBF) by the corresponding renal cytokine gradient (RV-IVC) for each measured product [47].

### 2.6. Cell Culture

The effect of ELAM on normal cells and its ability to decrease renal cellular injury was also studied in vitro. Pig proximal tubular epithelial (PK1) cells were cultured in Medium-199 (Gibco BRL) supplemented with 3% fetal bovine serum (FBS) and 1% antibiotics (100 U/mL penicillin and 100 μg/mL streptomycin) at 37 °C in a humidified atmosphere with 5% CO_2_ [48]. The culture medium was replaced every 2 days to remove non-adherent cells. At about 80–90% confluence, cells were digested and cultured overnight in 6-well plates. Then, the medium was changed to a fresh medium containing 3% FBS and different treatments.

PK1 cells were cultured for 24 h with or without 10 ng/mL TNF-α [49,50] and 10 mM palmitic acid (PA) [51,52], a model that mimics renal injury and lipotoxicity in vitro. Injured PK1 cells were treated with or without ELAM (1 nM for 6 h) [38,53], and then harvested for subsequent assays.

### 2.7. TMRE, MitoSOX and Western Blot

Mitochondrial membrane potential was measured in PK1 cells by tetramethylrhodamine ethyl ester (TMRE) staining (50 nM for 20 min at 37 °C, Thermo-Fisher T669, Waltham, MA, USA) [54], and mitochondrial ROS production was assessed by MitoSOX (2 μM for 30 min at 37 °C, Thermo-Fisher M36008) [25].

Protein concentration was measured with Bradford Protein Assay. Specific antibodies against mTOR, ULK-1, ATG-5-12, LC3 I/II, p-AMPK, AMPK, p-AKT, AKT, p-S6, S6 and GADPH were used as mentioned above. The density of each band was analyzed byAlphaView SA software (Cell Biosciences, Santa Clara, CA, USA).

### 2.8. Statistical Analysis

Statistical analysis was performed by IBM SPSS statistics software version 27.0 (Armonk, NY, USA). Normality was tested with GPower version 3.1 (Heinrich Heine University Düsseldorf, Düsseldorf, Germany). Results are presented as mean ± SD for normally distributed data and one-way analysis of variance (ANOVA) was followed by an unpaired student’s *t*-test. Non-normally distributed data were expressed as median (range) and compared with non-parametric tests (Wilcoxon and Kruskal–Wallis). A significant difference was assumed at *p* ≤ 0.05.

## 3. Results

Upon study conclusion at 16 weeks of diet, body weight and levels of fasting insulin, HOMA-IR scores, total cholesterol, HDL, and triglycerides were elevated in MetS compared to lean pigs (Table 1), consistent with development of MetS, which was unaffected by ELAM treatment. Serum creatinine levels were similar among the groups.

### 3.1. MetS Affects Autophagy

**Figure 1:**
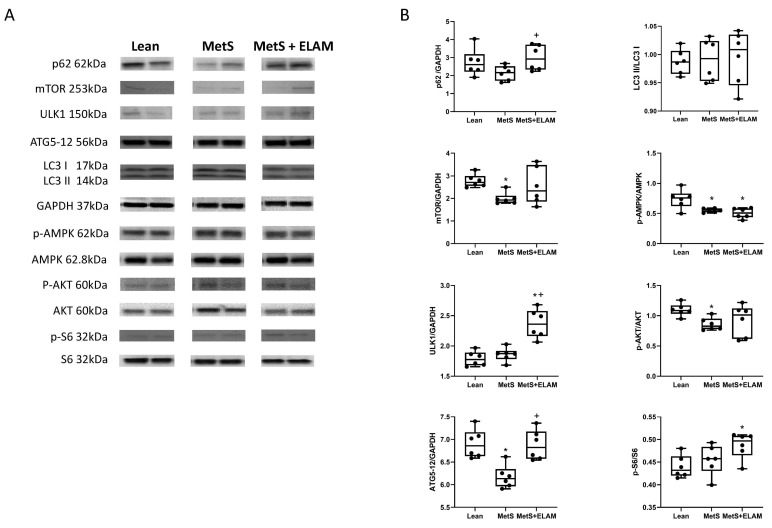
Renal protein expression of autophagy mediators. (**A**,**B**) mTOR, ATG5-12, AMPK, and P-AKT expression decreased in MetS kidneys and improved after ELAM therapy. p62 and ULK-1 also increased in MetS + ELAM kidneys. * *p* < 0.05 vs. lean, + *p* < 0.05 vs. MetS.

To assess the degree of renal cell autophagy, protein expression of p62, the ratio of LC3 II/LC3I, mTOR, AMPK, ULK1, and ATG5-ATG12 complex were measured in renal homogenates (Figure 1A). Expression levels of ATG5-ATG12 complex, mTOR, and p-AMPK were significantly downregulated in MetS vs. lean kidneys (Figure 1B, *p* = 0.006, *p* = 0.03, and *p* = 0.017, respectively). ULK-1 and ATG5-12 increased in MetS + ELAM (*p* = 0.001, *p* = 0.002 vs. MetS) and mTOR expression showed a strong trend to increase (*p* = 0.07 vs. MetS), whereas p-AMPK was unaffected (*p* = 0.23 vs. MetS, Figure 1B). MetS also tended to downregulate p62 expression (*p* = 0.058 vs. lean), which ELAM significantly elevated (*p* = 0.015 vs. MetS). There was no change in the LC3-II/LC3I ratio in either MetS (*p* = 0.86 vs. lean) or MetS + ELAM (*p* = 0.87 vs. MetS) kidneys. A decrease in Akt activity in MetS which might favor apoptosis was not reversed after ELAM treatment. The expression of p-S6 was unchanged in MetS (*p* = 0.39 vs. Lean) but increased by ELAM treatment (*p* = 0.01 vs. lean, *p* = 0.10 vs. MetS).

**Figure 2:**
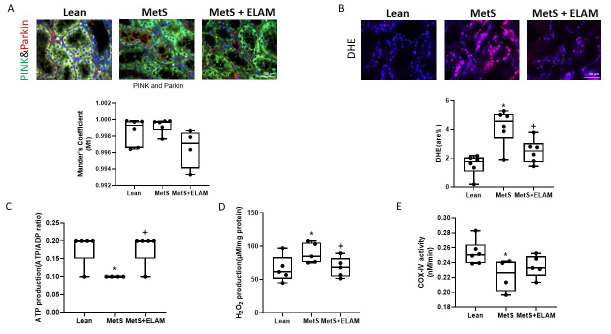
ELAM ameliorates mitochondrial function. (**A**) Representative renal immunofluorescence staining for PINK (green) and Parkin (red). No change in their co-localization showed no mitophagy. (**B**) Renal oxidative stress was evaluated by in situ production of superoxide anion by fluorescence microscopy using dihydroethidium (DHE), showing increased renal oxidative stress that ELAM ameliorated. (**C**–**E**) Mitochondrial function in MetS kidneys. Mitochondrial ATP production (ATP/ADP ratio) decreased in MetS compared to lean kidneys and were restored in MetS + ELAM. H_2_O_2_ production increased in MetS kidneys but normalized in MetS + ELAM. Compared to Lean, COX-IV activity was lower in MetS, but not in MetS + ELAM. * p < 0.05 vs. lean, *+ p <* 0.05 vs. MetS, Scale bar = 20 µm.

The colocalization (M1 factor) of renal Parkin and its co-localization with PINK were similar in MetS compared to the other two groups, arguing against an extension of the observed autophagy to mitophagy (Figure 2A).

### 3.2. Elamipretide Ameliorates MetS-Induced Cellular Mitochondrial Damage and Apoptosis

The renal superoxide anion production increased significantly in MetS compared with lean but was restored in MetS + ELAM (Figure 2B). Mitochondria isolated from the vehicle-treated MetS pigs exhibited lower production of ATP (ATP/ADP ratio) than either lean or MetS + ELAM pigs (both *p* = 0.024) (Figure 2C). MetS was also associated with increased mitochondrial oxidative stress, evidenced by a higher level of H_2_O_2_ production by mitochondria isolated from MetS kidneys (*p* = 0.034 vs. lean), which ELAM therapy reduced (*p* = 0.027 vs. MetS) (Figure 2D). Furthermore, COX-IV activity decreased in MetS compared to lean kidneys (Figure 2E, *p* = 0.046), consistent with mitochondrial dysfunction.

**Figure 3:**
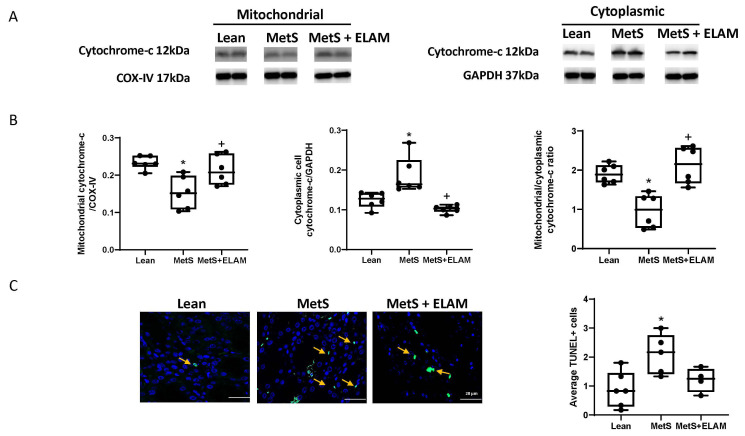
The degree of renal cell apoptosis in MetS was assessed by: (**A**,**B**) Cytochrome-c expression in mitochondrial compared to the cellular fraction. (**C**) TUNEL staining (yellow arrows showing apoptotic signals); Apoptosis increased in MetS and partly decreased in MetS + ELAM, suggesting improved mitochondrial function and cell survival. * *p* < 0.05 vs. lean, + *p* < 0.05 vs. MetS. Mean and median are shown as horizontal lines and accent marks, respectively. Scale bar = 20 µm.

Mitochondrial cytochrome-c expression decreased in MetS, whereas its cytoplasmic fraction increased (Figure 3A,B). Therefore, the ratio of mitochondrial-to-cytoplasmic cytochrome-c expression was much lower in MetS (Figure 3A,B, *p* = 0.0023) than in lean kidneys, suggesting mitochondrial release of cytochrome-c, consistent with apoptosis. However, ELAM significantly increased this ratio compared to MetS (*p* = 0.0004).

TUNEL staining revealed increased apoptotic signals within MetS kidneys (*p* = 0.011 vs. lean) which was partly mitigated by ELAM (*p* = 0.081 vs. MetS, Figure 3C) and no longer differed from lean.

### 3.3. Elamipretide Attenuates Renal Fibrosis and Injury

**Figure 4:**
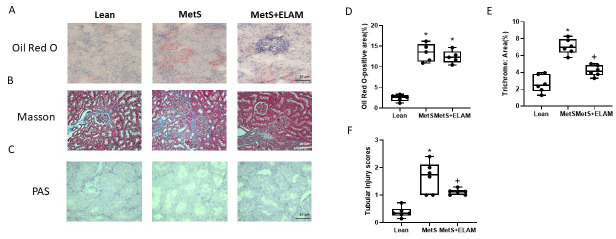
ELAM ameliorates renal damage in MetS. (**A**,**D**) Oil-red-O staining indicating kidney fat deposits in MetS, which were unaltered by ELAM; (**B**,**E**) Renal fibrosis (trichrome staining, blue) developed in MetS kidneys and decreased after ELAM. (**C**,**F**) Periodic acid-Schiff (PAS) stains showed increased renal tubular injury in MetS, which was reduced by ELAM. * *p* < 0.05 vs. lean, + *p* < 0.05 vs. MetS, Scale bar = 20 µm.

Oil-Red-O kidney staining revealed that, regardless of treatment, MetS was associated with greater fat deposits than lean pigs (*p* < 0.0001, Figure 4A,D). Trichrome staining showed obvious higher fibrosis in MetS compared to lean kidneys (*p* < 0.0001, Figure 4B,E), which was significantly attenuated in MetS + ELAM (*p* = 0.0001). Periodic acid-Schiff stains showed elevated renal tubular injury in MetS (*p* < 0.0001 vs. Lean, Figure 4C,F), which was reduced by ELAM (*p* = 0.0433 vs. MetS).

### 3.4. Elamipretide Attenuates MetS-Induced Renal Inflammation

**Figure 5:**
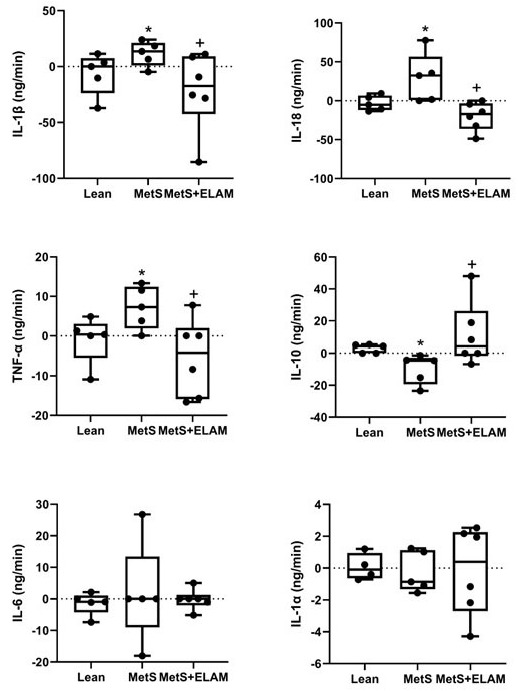
Renal production of inflammatory markers. The renal net release of the inflammatory cytokines IL-1β, IL-18, and TNF-α increased in MetS and decreased after ELAM treatment, whereas IL-10 decreased in MetS pigs and improved in MetS + ELAM pigs. * *p* < 0.05 vs. lean, + *p* < 0.05 vs. MetS.

The renal net release of the inflammatory markers IL-1ß, IL-18, and TNF-α was higher in MetS compared with Lean groups (*p =* 0.05, *p =* 0.029, and *p =* 0.028, respectively), but decreased after treatment with ELAM (*p =* 0.039, *p =* 0.005, and *p =* 0.015 vs. MetS, respectively, Figure 5). In contrast, renal release of the anti-inflammatory cytokine IL-10 was lower in MetS compared to Lean pigs (*p =* 0.010, Figure 5), but ELAM therapy increased its levels (*p =* 0.029 vs. MetS, Figure 5).

Net renal release of IL-1α and IL-6 remained unchanged among the groups (Figure 5).

### 3.5. ELAM Attenuates Mitochondrial Dysfunction in Injured PK1 Cells

**Figure 6:**
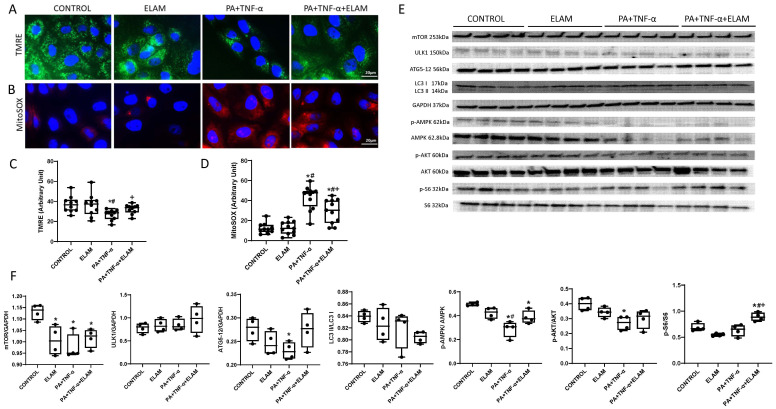
ELAM improves injured-PK1 cells mitochondrial function and autophagy proteins. (**A**,**B**) representative immunofluorescence staining (×40) for the mitochondrial membrane potential marker tetramethylrhodamine ethylester (TMRE; green) and the mitochondrial superoxide indicator (MitoSOX; red). (**C**,**D**) mitochondrial membrane potential decreased in PA+TNF-α-treated cells compared with control but increased after ELAM. Mito-SOX showed an opposite pattern in mitochondrial oxidative stress. (**E**,**F**) Protein expression of autophagy mediators in PA+TNF-α-treated PK1 cells with or without ELAM. * *p* < 0.05 vs. CONTROL, # *p* < 0.05 vs. ELAM, + *p* < 0.05 vs. PA+TNF-α. Scale bar = 20 µm.

In our MetS animal model, both renal injury and lipid overload coexisted. Therefore, we choose TNF-α to mimic renal injury and palmitic acid to mimic renal lipotoxicity. Mitochondrial membrane potential decreased in PA+TNF-α-injured cells (*p* = 0.023 vs. control), but increased after ELAM (*p* = 0.020 vs. PA+TNF-α, Figure 6A,C). Production of mitochondrial ROS, which increased in injured PK1 cells (*p* < 0.0001 vs. control), decreased, although was not normalized in the ELAM-treated injured PK1 cells (*p* = 0.0063 vs. PA+TNF-α, Figure 6B,D). ELAM alone had no influence on mitochondrial function in PK1 cells compared to controls (Figure 6A–D).

### 3.6. Autophagy in PA+TNF-α-Injured PK1 Cells

PA+TNF-α exposure decreased protein expression of mTOR and ATG5-12 compared with control (Figure 6E,F), but did not affect ULK-1 or LC3 II/I. The p-AMPK/AMPK ratio decreased after PA+TNF-α, and ELAM improved although not fully normalized it. After ELAM treatment in injured-PK1, p-AKT/AKT was no longer lower than control (Figure 6E,F). Furthermore, ELAM up-regulated the expression of p-S6/S6 (Figure 6E,F).

## 4. Discussion

This study suggests that autophagy is slightly attenuated in the kidneys of pigs with diet-induced metabolic syndrome, concomitant with elevated renal apoptosis, inflammation, and fibrosis. These phenomena are accompanied by a fall in mitochondrial energy production and an increase in oxidative stress, as indicated by a higher rate of mitochondrial H_2_O_2_ production. Treatment with a specific mitochondrial-targeted peptide improved mitochondrial function, restored autophagy, and decreased inflammation, consistent with a link between these processes, and ultimately improved renal structure. These findings suggest a role for mitochondria and cellular autophagy in renal protection in MetS.

MetS causes fat deposition and lipid oxidation in kidney cells [55], resulting in intraglomerular hypertension, podocyte damage, and possibly segmental sclerosis [56] due to increased rate of cellular apoptosis [57]. We have previously shown that development of MetS can also impact cardiomyocyte autophagy, which was attenuated during the evolution of obesity to MetS [40]. Increased oxidative stress and damage to mitochondria result in augmented apoptosis accompanied by attenuated autophagy [58], a scenario that ultimately becomes maladaptive and inhibits repair of kidney parenchyma.

An evolutionary-conserved mechanism, autophagy involves different proteins working as cogwheel elements to respond to stress and eliminate redundant elements. Autophagy maintains tubular cell integrity under both physiological conditions as well as stress conditions such as ischemic injury or aging [59]. Upon nutrient abundance, increased stress is initially sensed and autophagy triggered by AMPK activation (phosphorylation) and upregulation of mTOR expression [60]. Autophagy is then initiated by cooperation between ATG5 and 12 adhesion and ULK1 [61]. For example, in proximal tubular cells, cellular stress may upregulate ATG5 leading to increased autophagy [62]. The ULK1 complex, an essential regulator of mammalian autophagy, can be directly phosphorylated and activated by AMPK [63,64]. Interestingly, ULK1 was unchanged in our MetS model, suggesting an unaltered initiation phase of autophagy. Contrarily, lower ATG5-12 expression in MetS indicates an elongation error. Lower p-AMPK, as well as mTOR (which activates autophagy), further highlights this deficient starting phase of autophagy in MetS. AKT may inhibit autophagy through Beclin-1 inactivation [65], and our studies also revealed decreased p-AKT expression in MetS. Downregulation of these proteins in MetS pig kidneys is therefore consistent with a lower rate of early autophagy.

On the other hand, our findings may suggest that some elements of autophagy remained intact in our model, suggesting that this impairment was mild. P-S6Ser240/244, a marker of mTORC1 activation and master regulator of autophagy [66], was unaltered in MetS kidneys, yet increased after ELAM treatment, suggesting enhancement of autophagy. While increased S6 activity in MetS kidneys may imply that cells successfully sense the nutrient stress and instigate autophagy, downregulation of other vital components may impede its progression. Furthermore, we found a trend for lower p62 expression in MetS, whereas decreased autophagy often leads to its accumulation. However, notably, p62 expression is determined not only by autophagic degradation, but also by its transcriptional upregulation and by the availability of lysosomal-derived amino acids, and therefore does not necessarily faithfully reflect autophagic activity [67]. Possibly, the unchanged levels of p62 may be related to the early phase or short duration of Mets in our model, which is a weakness of our study. Additionally, LC3 plays a pivotal role in progression of autophagy. LC3-I conjugates to phosphatidylethanolamine to form LC3-II and recruit autophagosomes, and their ratio (LC3-I/LC3-II) thus often changes in conditions that alter autophagy [68]. In the early stages of MetS, if the core autophagosome forms, it can bind to the cellular membrane and progress to forming an autophagosome. The unchanged ratio of LC3-I and -II in MetS may, therefore, suggest intact autophagic flux. Nevertheless, most of the evidence implies at least partial autophagy malfunction in MetS, which might lead to accumulation of noxious components, eventuating in apoptosis [69]. PINK1 functions in mitochondrial maintenance and recruits Parkin (an E3 ubiquitin ligase) from the cytoplasm to damaged mitochondria with low membrane potential to induce mitophagy [70]. Here, we found that co-localization of PINK and Parkin did not change, arguing against extension of autophagy to mitophagy.

Importantly, besides affecting autophagy, we observed in MetS kidneys increased mitochondrial superoxide anion and H_2_O_2_ production accompanied by decreased ATP production and COX-IV activity. H_2_O_2_ may trigger opening of the mitochondrial permeability transition pore, eliciting cytochrome-c release to the cytoplasm and evoking apoptosis, which may account for accumulation of TUNEL-positive cells within MetS kidneys. Reduced ATP levels imply functional impairment of mitochondria, which may fail to meet cellular energy requirements. In turn, lower ATP production reduces autophagy rates, especially in the context of nutrient imbalance such as MetS [71,72] or starvation. Healthy mitochondria are also potential sources of autophagosomal membrane and regulate mTOR and AMPK activities [73]. The reciprocal relationship between autophagy and mitochondria reflects in the observation that a reduction in autophagy (and mitophagy) in turn increases the number of defective mitochondria, accelerating pro-apoptotic signaling that may cause cell death [74].

Dyslipidemia can cause renal dysfunction [75] by several mechanisms. Kidney cells uptake fatty acids, common energy substrates, from capillaries by multiple cell surface lipid transport proteins, such as cluster of differentiation36 (CD36) [76]. Then, fatty acids activated to acyl-CoA in the cytosol are shuttled into the mitochondrial matrix through carnitine [77,78] that participates in the tricarboxylic acid cycle and ATP production [79]. Moreover, carnitine-acetyltransferase can transport acetyl-CoA out of mitochondria, forming phospholipids, cholesterol, and triacylglycerol through integrated endogenous lipid conversion [80,81]. However, lipid overload can instigate kidney ER stress and mitochondria dysfunction [82,83], interfere with protective autophagy, and eventuate in cell damage. The notion of “lipid nephrotoxicity” is underscored by evidence of renal lipid accumulation in patients with CKD [84,85]. Additionally, hyperlipidemia influences mitochondrial metabolism, resulting in excessive ROS production, apoptosis, and kidney fibrosis [85,86]. Pertinently, similar processes may occur in multiple organs and underlie multi-organ crosstalk in dyslipidemia and MetS.

The central role of mitochondria in renal metabolic abnormalities positions them as key therapeutic targets in MetS. ELAM stabilizes cardiolipin and preserves the mitochondrial inner membranes [87,88], thereby decreasing the rate of apoptosis [89]. We have previously shown that MetS decreased renal content of the inner mitochondrial membrane cardiolipin, particularly the tetra-linoleoyl (C18:2) species, and ELAM normalized its content and remodeling [33]. The current study implies that ELAM also reinstates autophagy in vivo, possibly by restoring bioenergetics and preventing oxidative damage to proteins, which might in turn improve stress tolerance, attenuate apoptosis, and reduce structural damage to the MetS kidney [32,90]. Our in vitro studies suggest that ELAM may contribute to improved mitochondrial function by restoring membrane potential and blunting mitochondrial production of ROS, and also reinstates autophagy in PK1 cells injured using PA+TNF-α. Pertinently, LC3 contains specific cardiolipin-binding sites required for autophagy activation [91]. Yet, ELAM targets the mitochondria specifically, with no direct effects on autophagy [92]. Hence, its attenuation in MetS + ELAM implicates mitochondrial injury in the mechanism of abnormal autophagy observed in MetS. Non-recycled components may cause cellular malfunction in stress states [93]. Identifying methods to activate autophagy may therefore have implications for other diseases involving deficient autophagy.

Mitochondrial dysfunction and autophagy may modulate or result from inflammatory processes. Kidney damage can be induced by inflammatory cytokines such as IL-1β, which is activated by the NLR family pyrin domain-containing-3 (NLRP3) inflammasome to enhance inflammatory cell infiltration and adhesion molecule expression [94,95]. Notably, blunted autophagy may exacerbate diabetes-induced podocyte damage, thereby worsening nephropathy [96]. Here, we found that MetS increased renal release of pro-inflammatory cytokines (IL-1β, IL-18, and TNF-α), which ELAM treatment remarkably attenuated. Congruently, the release of anti-inflammatory IL-10 was increased after injections of ELAM as did autophagy, suggesting that its protective effects in the MetS kidney involved anti-inflammatory and pro-autophagy mechanisms.

Studies indicate that autophagy can protect cells through several anti-inflammatory mechanisms. Autophagy may degrade the NLRP3 inflammasome, preventing the production of proinflammatory cytokines including IL-1β and IL-18 [97], and reducing activation of the proinflammatory transcription factor nuclear-factor of kappa light polypeptide gene enhancer in B-cells (NF-κB) [98]. Importantly, basal autophagy also inhibits IL-1β secretion, clears cytoplasmic dysfunctional mitochondria, and thereby prevents accumulation of mitochondrial ROS that activate the inflammasome [99,100]. Indeed, autophagy inhibition increases mitochondrial fission and cell death [101]. Conversely, the depolarized and damaged mitochondria may accumulate and leak endogenous inflammasome agonists, such as mitochondrial DNA and ROS [99,100]. Therefore, by protecting mitochondria, ELAM might have both decreased mitochondrial ROS production, restored the electron-transport-chain [102], blunted inflammation, and permitted autophagy, thereby interrupting the vicious cycle of autophagy, mitochondrial damage, and inflammation, and protecting the MetS kidney.

Our study is limited by the relatively small group size, due to the labor and cost of porcine disease models, and the short duration for which pigs were exposed to MetS, which might account for the relatively subtle changes in autophagy indices. This is a notable weakness of our model. On the other hand, large animals remain a largely untapped resource. Given the similarities in pathophysiology to humans for exploring disease etiology and prevention, our model mimics the human condition of early Mets and may thus afford clinical translation. Furthermore, the expression of several autophagy-related proteins did not show a statistically significant change in MetS, suggesting that the impairment in autophagy was mild. Yet, some of the altered proteins can account for subtle changes in autophagy. Follow-up studies are needed to confirm changes in RNA expression of target autophagy proteins and the connection between mitochondrial dysfunction and deregulated autophagy. Future studies will also determine if different timing or duration of MetS elicits a more complete blockade of autophagy. A control group of normal pigs treated with ELAM was unavailable, but in previous studies, we found no effect of ELAM on healthy pig kidneys [39], and our in vitro studies revealed no measurable effects of ELAM on normal PK-1 cells. In addition, ELAM did not restore systemic parameters or AMPK, an important metabolic sensor, likely because lipid levels remained unchanged. Thus, the impact of ELAM on the preservation of kidney function in pigs is probably direct and not mediated via systemic characteristics.

## 5. Conclusions

Renal cellular stress in the context of MetS slightly reduces levels of autophagy, while increasing apoptosis and fibrosis, a process essential to maintaining cell homeostasis. Treatment with the mito-protecting drug ELAM improved mitochondrial energy production, decreased production of hydrogen peroxide, and restored autophagy partly, thereby decreasing renal apoptosis, inflammation, and fibrosis. Thus, decreased autophagy and mitochondrial protection might be therapeutic targets to prevent kidney injury in MetS.

## Figures and Tables

**Table 1:** Systematic parameters in pigs with a 16-week metabolic syndrome (MetS) untreated or treated with elamipretide (ELAM).

Parameter	Lean	Mets	Mets + ELAM
Body weight (Kg)	68 ± 6.0	92.3 ± 4.3 *	93.1 ± 3.4 *
Glucose (mg/dL)	111 ± 3.5	108.1 ± 26.5	109.3 ± 24.3
Fasting Insulin (µU/mL)	0.4 ± 0.008	0.9 ± 0.2 *	0.7 ± 0.1 *
HOMA-IR score	0.6 ± 0.1	1.3 ± 0.2 *	1.2 ± 0.1 *
Total cholesterol (mg/dL)	72.3 ± 6.9	564.2 ± 68.3 *	603.0 ± 62.3 *
HDL cholesterol (mg/dL)	42.9 ± 1.4	164.8 ± 28.1 *	130.3 ± 9.8 *
LDL cholesterol (mg/dL)	29.1 ± 2.7	389.1 ± 48.9 *	390.3 ± 50.1 *
Triglycerides (mg/dL)	7.4 ± 0.6	12.5 ± 2.1 *	13.2 ± 1.8 *
Creatinine (mg/dL)	1.67 ± 0.24	1.45 ± 0.20	1.62 ± 0.40

HOMA-IR: hemostasis model assessment of insulin resistance, HDL: high-density lipoprotein, LDL: low-density lipoprotein, * *p* < 0.05 vs. lean.

## Data Availability

Data are available from the corresponding author upon reasonable request.
